# Suvorexant, a Dual Orexin Receptor Antagonist, Protected Seizure through Interaction with GABA_A_ and Glutamate Receptors

**DOI:** 10.22037/ijpr.2019.14688.12584

**Published:** 2020

**Authors:** Bibi Marjan Razavi, Omid Farivar, Leila Etemad, Hossein Hosseinzadeh

**Affiliations:** a *Targeted Drug Delivery Research Center, Pharmaceutical Technology Institute, Mashhad University of Medical Sciences, Mashhad, Iran. *; b *Department of Pharmacodynamics and Toxicology, School of Pharmacy, Mashhad University of Mdical sciences,Mashhad,Iran. *; c *Department of Pharmacodynamics and Toxicology, School of Pharmacy, Mashhad University of Medical Sciences, Mashhad, Iran.*; d *Pharmaceutical Research Center, Pharmaceutical Technology Institute, Mashhad University of Medical Sciences, Mashhad, Iran.*

**Keywords:** Suvorexant, Orexin, Anti-epileptic, Anti-seizure, GABA_A_, NMDAR, AMPAR

## Abstract

Orexin can increase neuronal excitability and induce epileptic activity. In this study, the effects of suvorexant (orexin receptor antagonist) on pentylenetetrazol (PTZ) and maximal electroshock (MES)-induced seizure were investigated. Mice were divided into 5 groups of six animals each including normal saline (10 mL/kg), diazepam (2 mg/kg), and suvorexant (50, 100 and 200 mg/kg) groups. In PTZ test, the latency to first minimal clonic seizure (MCS), latency to the first generalized tonic–clonic seizures (GTCS), total duration of seizure and also protection against mortality were evaluated. In MES, the hind limb tonic extension (HLTE) and the protection against mortality were recorded. In order to evaluate the role of GABA_A_ in anticonvulsant effect of suvorexant, flumazenil was used and to investigate the role of glutamate, the protein levels of AMPAR and NMDAR were measured in hippocampus by western blotting.  In PTZ model, suvorexant (200mg/kg) increased MCS and GTCS latencies. Suvorexant (100 and 200 mg/kg) decreased total duration of seizure compared to control group. In PTZ model, flumazenil inhibited the prolongation of seizure latency induced by suvorexant. In MES, the HLTE was decreased by suvorexant (100 and 200 mg/kg) and suvorexant was protected against mortality by 83.3%. Moreover, the protein levels of NMDAR and AMPAR were decreased by suvorexant.

Suvorexant exerted anticonvulsant activity and in addition to its inhibitory effect on orexin receptors, this effect may be mediated, at least partly, through interaction with GABA_A_ and glutamate receptors.

## Introduction

Epilepsy is a neurological disorder which is associated with recurrent seizures. Excessive discharge in hyperexcitable neurons leads to epileptic seizures. Although, most epileptic seizures result from discharging originated in cortical and hippocampal structures, some types of seizures was generated in subcortical structures (1). It is estimated that 50 million individuals of all ages have a diagnosis of epilepsy in the world (2). Although, there are many anti-epileptic drugs for the treatment of epilepsy, different side effects and resistance to treatment in almost 30 % of people have been observed (3). 

Orexin-A and Orexin-B (hypocretin-1 and -2) are 33 and 28 amino acid neuronal polypeptides, which are considered as internal ligands of a G-coupled orphan receptor in the hypothalamus. The orexin peptides bind selectively to the OX1 (orexin-1) and OX2 (orexin-2) receptors (OX1R and OX2R). Orexin-containing cell bodies are located in the lateral and perifornical regions of the hypothalamus, but their fibers project broadly throughout the central nervous system (4). The role of orexinergic system in different physiological processes such as arousal, reward seeking behavior, energy homeostasis, sensory modulation, stress processing, locomotion, cognition, endocrine functions, visceral functions, and pain modulation has been established (5, 6).

It has been suggested that orexins are involved in the pathophysiology of epilepsy (7). For example, a high dose of orexin-A induced seizure-related behaviors after intracerebroventricular (ICV) administration in rats (8). Moreover, the intracortical injection of orexin-A and orexin-B induced seizure (9) and increased penicillin-induced epileptic activity (10). Furthermore, a recent study showed that intra hippocampal administration of orexin receptor antagonists has anti-epileptic activity through the alteration of GABA and glutamate levels (11). In another study, the development of epilepsy was prevented by inhibiting orexin receptors that was associated with neurogenesis and sleep suggesting that orexin receptor antagonists have anti-epileptic activity (7). Moreover, in another study, an orexin-A receptor antagonist (SB-334867) protected 4-aminopyridine (4-AP)-induced seizure-like activities in anesthetized rats (12).

Suvorexant, a dual orexin receptor antagonist, has the ability to treat insomnia. In May 2013, suvorexant was approved by Food and Drug Administration (FDA) for the treatment of patients with sleep difficulties (13). In the present study, the efficacy of suvorexant, as an orexin receptor antagonist, on pentylenetetrazol (PTZ) and maximal electroshock (MES)-induced seizure has been evaluated.

## Experimental

*Animals*

 Male albino mice (weighing 25 ± 3 g) have been used in this study. The animals were housed in a ventilated room under a 12/12-hour light/dark cycle at 24 °C with free access to water and food. All animals in these experiments were carried out in accordance with Mashhad University of Medical Sciences, Ethical Committee Acts (No: 941662).

*Chemicals*

 Suvorexant was purchased from Trademax Pharmaceuticals & Chemicals CO, China. Flumazenil and pentylenetetrazol were provided from Sigma-Aldrich and Merck, respectively. 

*Study design*

 The mice were randomly divided into 5 groups: 1- negative control (normal saline plus carboxymethyl cellulose, gavage), 2- positive control (diazepam, 2 mg/kg, IP.) (14), 3, 4 and 5-suvorexant (50, 100, and 200 mg/kg, gavage, dissolved in carboxymethyl cellulose) (15). To evaluate the roles of GABAergic and glutamatergic systems in anticonvulsant effect of suvorexant, flumazenil (10 mg/kg), an antagonist of benzodiazepine (BZD) site in the GABA_A_-BZD receptor complex was administrated 30 min prior to suvorexant (200 mg/kg), negative and positive controls (16). In another set of experiment, suvorexant (200 mg/kg) was administrated to mice for 7 days. After the induction of seizure, the mice were killed and hippocampi were dissected. The protein levels of NMDAR and AMPAR were measured by western blot analysis.

*Anticonvulsant activity*

*Pentylenetetrazol (PTZ)-induced seizure*

Suvorexant, negative, and positive controls were administrated 60 min prior to PTZ (80 mg/Kg, IP). The animals were placed individually in plastic boxes and observed for 20 min. In PTZ model, the latency to the first minimal clonic seizure (MCS), latency to the first generalized tonic–clonic seizures (GTCS), the total duration of seizures, and protection against mortality were evaluated (14). 

*Maximal electroshock seizure (MES) test*

Suvorexant, negative, and positive controls were administrated 60 min prior to the MES test. Then, a stimulus train was applied via ear-clip electrodes (sinusoidal pulses, 120 mA and 60 Hz, for 0.2 seconds) using a constant current stimulator (Digital Electroshock Model 150, Eghbal Teb Co., Mashhad, Iran). A drop of 0.9% saline solution was applied on each ear of the animal prior to placing the electrode. The duration of hind limb tonic extension (HLTE), and the protection against mortality were recorded (14).

*Western blotting*

For western blot analysis, the hippocampus was dissected from the brains of the mice. Tissues were homogenized at 4 °C in the lysis buffer containing 50 mM Tris–HCl, pH 7.5, 2 mM EDTA, 2 mM EGTA , 10 mM sodium-β glycerophosphate, 0.1% (w/v) SDS, 1% (v/v) Triton X-100 , 1 mM sodium orthovanadate, 10 mM NaF, 0.1% (v/v) 2-mercaptoethanol (Sigma, cat#S6508, Germany), 2% (w/v) sodium deoxycholate, 1 mM phenylmethylsulfonyl fluoride (PMSF), and 2 µl complete protease inhibitor cocktail using a Polytron homogenizer (IKA^®^T10, Germany). Then, the lysates were sonicated on ice using a probe sonicator (UP100H, Germany). After centrifugation at 10000 g for 10 min at 4 °C, the supernatants were collected and transferred to clean microtubes and the protein concentrations were determined using a Bio-Rad protein assay kit. The supernatants were mixed with equal volumes of 2X SDS buffer, containing 100 mM Tris-base, 20% v/v Glycerol, 4% w/v SDS, 10% v/v 2-mercaptoethanol and 0.2% w/v bromophenol blue, heated in a boiling water bath for 10 min. and stored at −80 °C. Briefly, equal amounts of total proteins from each sample were loaded onto a SDS polyacrylamide gel. After electrophoresis, the protein bands were transferred to a PVDF membrane. The membranes were blocked for 3 h in TBST (Tris-buffered saline with 0.5% Tween 20 containing 5% milk). Next, the blots were probed at room temperature with specific antibodies for 2 h. The membranes were washed three times for 5 min and incubated with HRP-conjugated secondary antibodies for 1–2 h. The primary antibodies were mouse polyclonal anti-serum against AMPA (GluR 2/3/4) (Cell signaling #2460), mouse polyclonal anti-serum against NMDA receptor (Cell signaling #4205) and mouse polyclonal anti-serum against β-actin (Cell signaling #3700). All antibodies were used at a dilution of 1:1000. Anti-mouse lgG labeled with horseradish peroxidase (Cell Signaling, #7076) was used as secondary antibody. Immuno-labelled bands were visualized using an ECL detection reagent kit (Pierce, cat # 32106, USA) and Alliance 4.7 gel doc (UK). Intensities of the protein bands were analyzed by optical densitometry using UV band image analysis software (UVITEC, Cambridge, UK). All bands were normalized against corresponding β-actin intensities.

*Statistical analysis*

 All results were expressed as mean ± SEM. One-way ANOVA followed by Tukey–Kramer test was performed to compare the means. P values less than 0.05 were considered as significant. Fisher’s exact test was used to compare protection against mortality between the groups.

## Result

*Antiepileptic activity of suvorexant in PTZ-induced seizure model*

**Figure 1:**
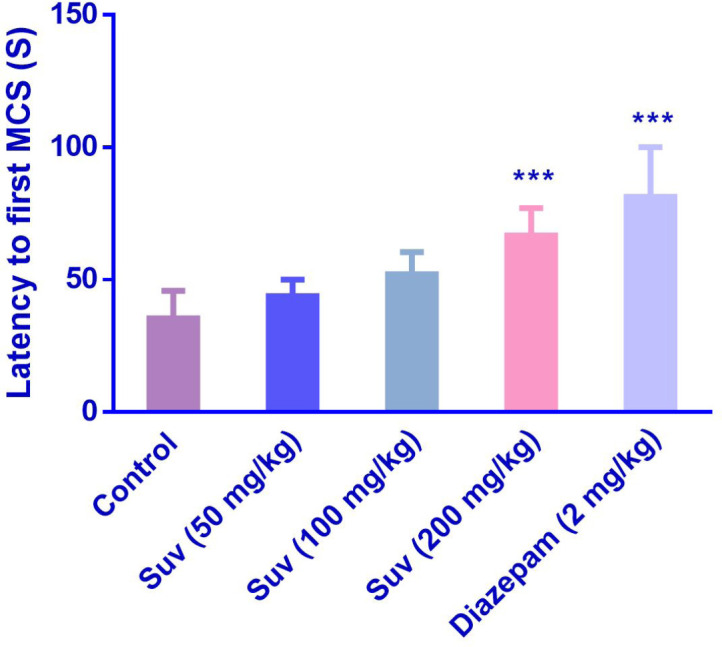
Effect of suvorexant on the latency to the first minimal clonic seizure (MCS) in PTZ-induced seizure in mice. Data are presented as mean ± SEM. Tukey Kramer, ^***^p < 0.001 vs control, n = 6

**Figure 2:**
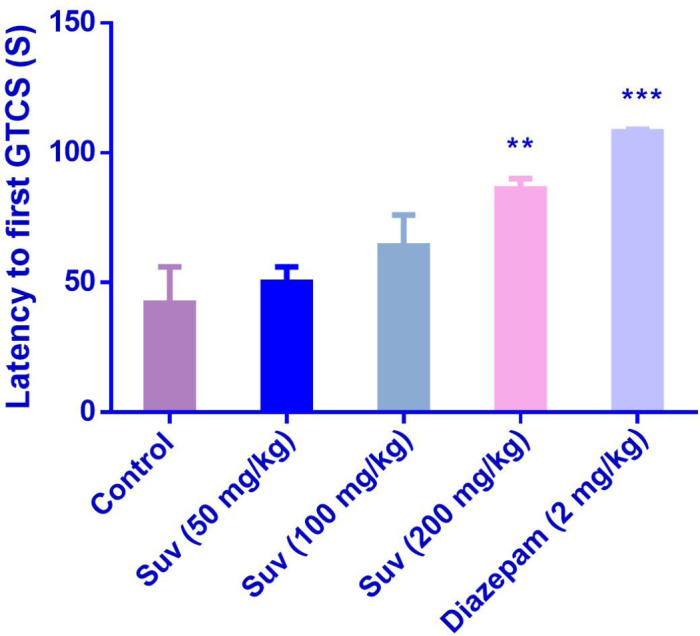
Effect of suvorexant on the latency to first generalized tonic-clonic seizure (GTCS) in PTZ-induced seizure in mice. Data are presented as mean ± SEM. Tukey Kramer, ^**^p < 0.01 and ^***^p < 0.001 vs control, n = 6

**Figure 3:**
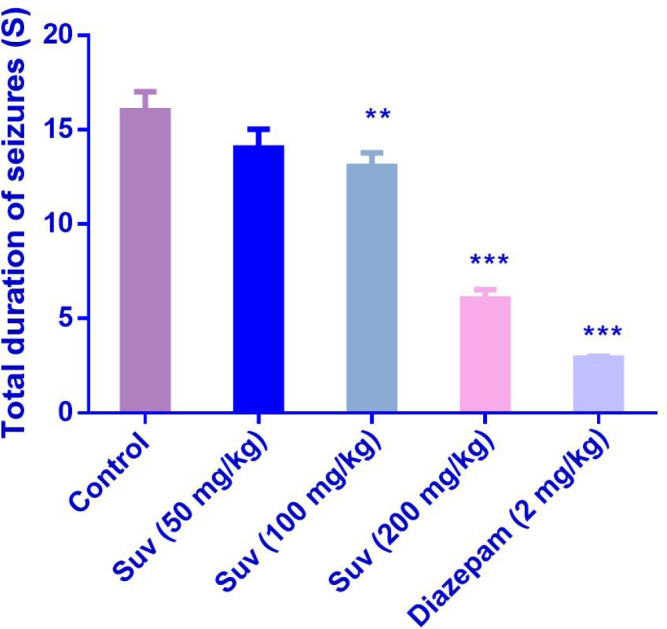
Effect of suvorexant on total duration of seizures in PTZ-induced seizure in mice. Data are presented as mean ± SEM. Tukey Kramer, ^**^p < 0.01 and ^***^p <0.001vs control, n = 6

Suvorexant (200 mg/kg) increased the latency to the first MCS (P<0.001) (Figure 1) and latency to the first GTCS (*P*<0.01) (Figure 2), compared to the negative control group. In figure 3, suvorexant (100 and 200 mg/kg) was able to reduce the total duration of seizure, compared to the negative control (*P *< 0.01 and *P *< 0.001). Suvorexant (100 and 200 mg/kg) could protect against mortality 83.3% (Table 1). 

*Antiepileptic activity of suvorexant in MES-induced seizure model*

**Figure 4:**
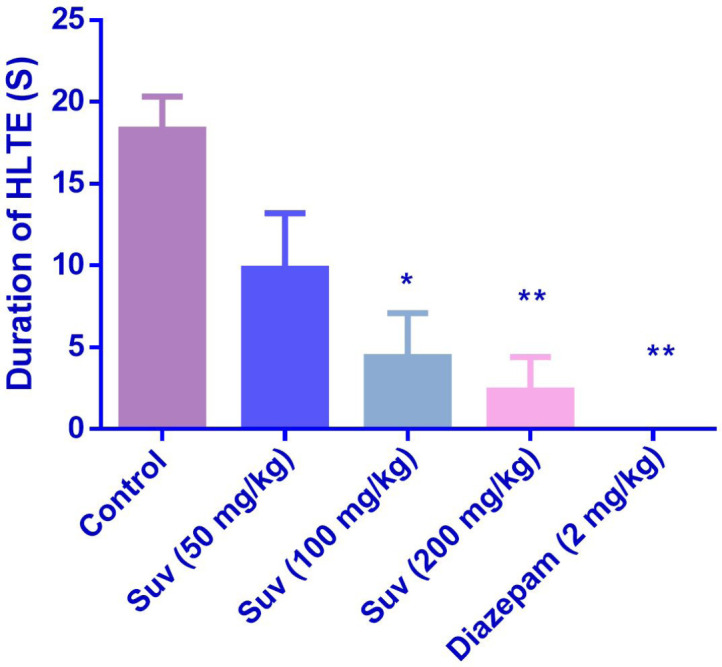
Effect of suvorexant on duration of hind limb tonic extension (HLTE) in MES-induced seizure in mice. Data are presented as mean ± SEM. Tukey Kramer, ^*^p < 0.05 and ^**^p <0.01 vs control, n=6

In the MES test, suvorexant (100 and 200 mg/kg) reduced the duration of HLTE (Figure 4) (*P *<0.05 and *P *< 0.01). Suvorexant (100 and 200 mg/kg) could protect against mortality 83.3% (Table 2). 

*The effect of flumazenil on the anticonvulsant activity of suvorexant*

**Figure 5:**
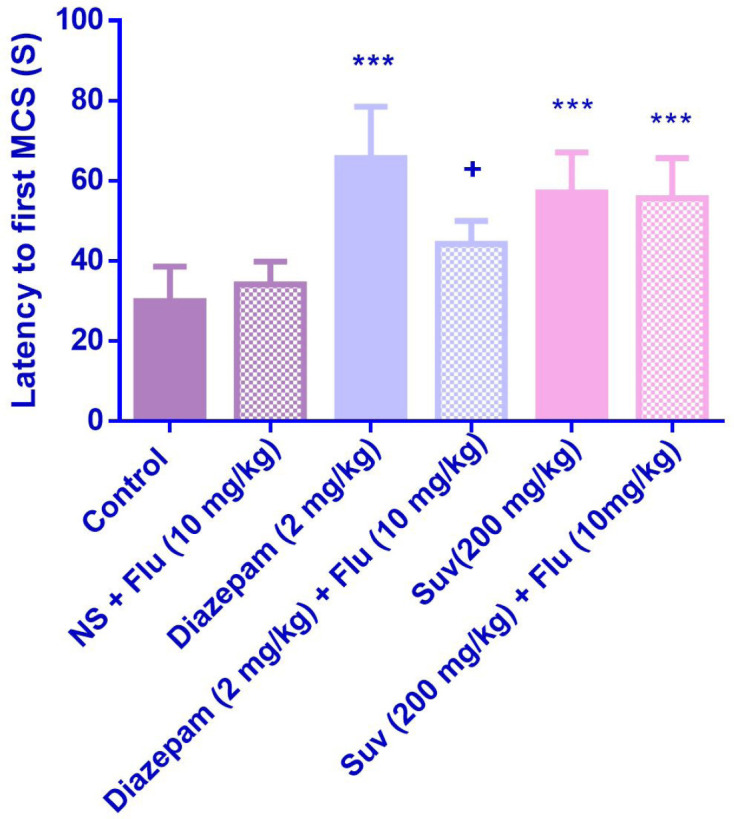
Effect of suvorexant on the latency to first minimal clonic seizure (MCS) in PTZ-induced seizure in the presence and absence of flumazenil in mice. Data are presented as mean ± SEM. Tukey Kramer, ***p < 0.001 vs normal saline, + *p *< 0.05 vs agent received flumazenil. n=6. (N.S=normal saline, Flu= flumazenil).

**Figure 6:**
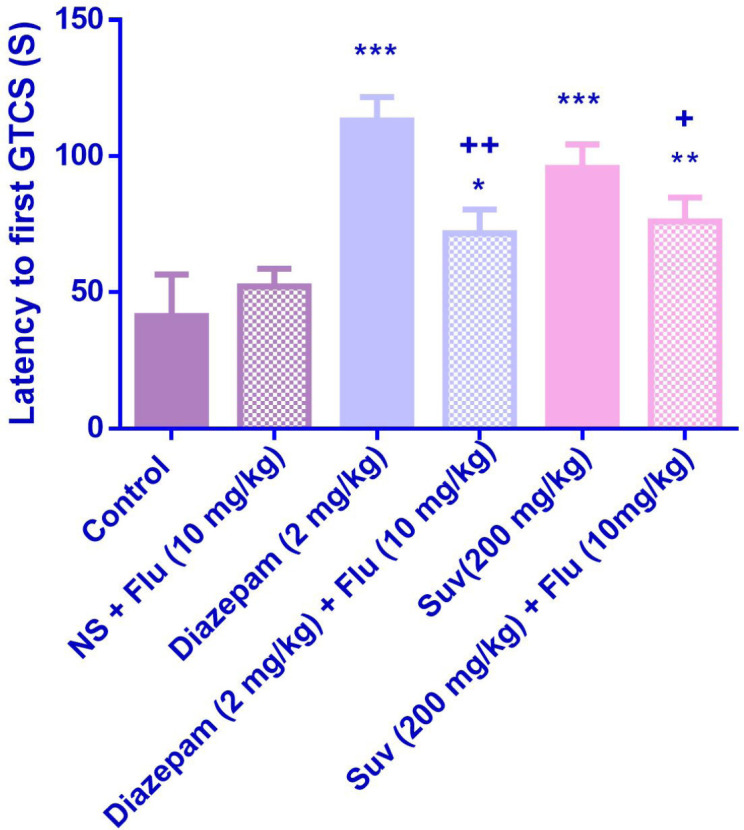
Effect of suvorexant on the latency to first generalized tonic-clonic seizure (GTCS) in PTZ-induced seizure in the presence and absence of flumazenil in mice. Data are presented as mean ± SEM. Tukey Kramer, *p < 0.05, **p < 0.01 and ***p < 0.001 vs normal saline, +*p *<0.05 and ++*p *< 0.01vs agent received flumazenil. N = 6. (N.S=normal saline, Flu= flumazenil).

Flumazenil reduced the anticonvulsant activity of suvorexant (200 mg/kg) by decreasing the latency to the first GTCS (*p* < 0.05) (Figure 6). Although flumazenil decreased the anticonvulsant activity of suvorexant by reducing the latency to the first MCS, the effect was not significant (Figure 5).

*The effect of suvorexant on AMPAR and NMDAR protein expressions in hippocampus*

**Figure 7:**
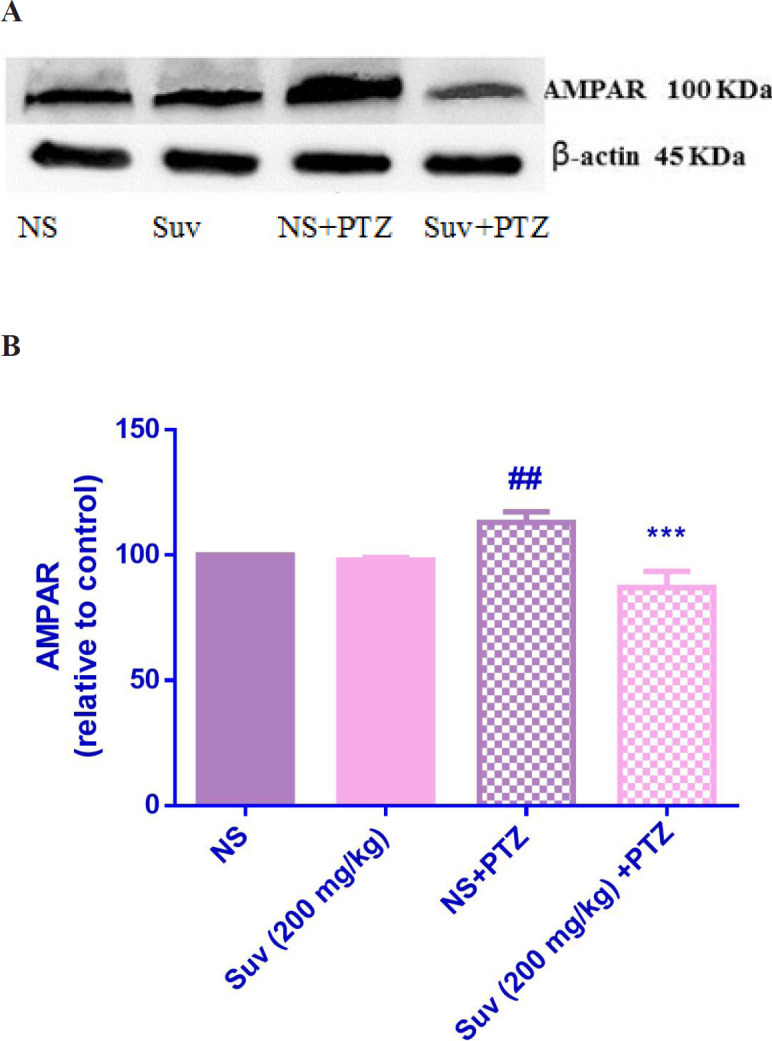
Effect of suvorexant on the protein level of AMPAR in the mice hippocampus. Suvorexant was administrated for seven days. After seizure induction by PTZ, hippocampus was dissected. Representative western blots showing specific bands for AMPAR and b-actin as an internal control. Equal amounts of protein sample (50 µg) obtained from whole hippocampus homogenate were applied in each lane. These bands are representative of four separate experiments. (B) Densitometric data of protein analysis. Data are expressed as the mean ± SEM. ##*p* < 0.01 vs normal saline, ***p < 0.001 vs PTZ+ normal saline. NS (normal saline), SUV(suvorexant), PTZ(pentylenetetrazol).

**Figure 8:**
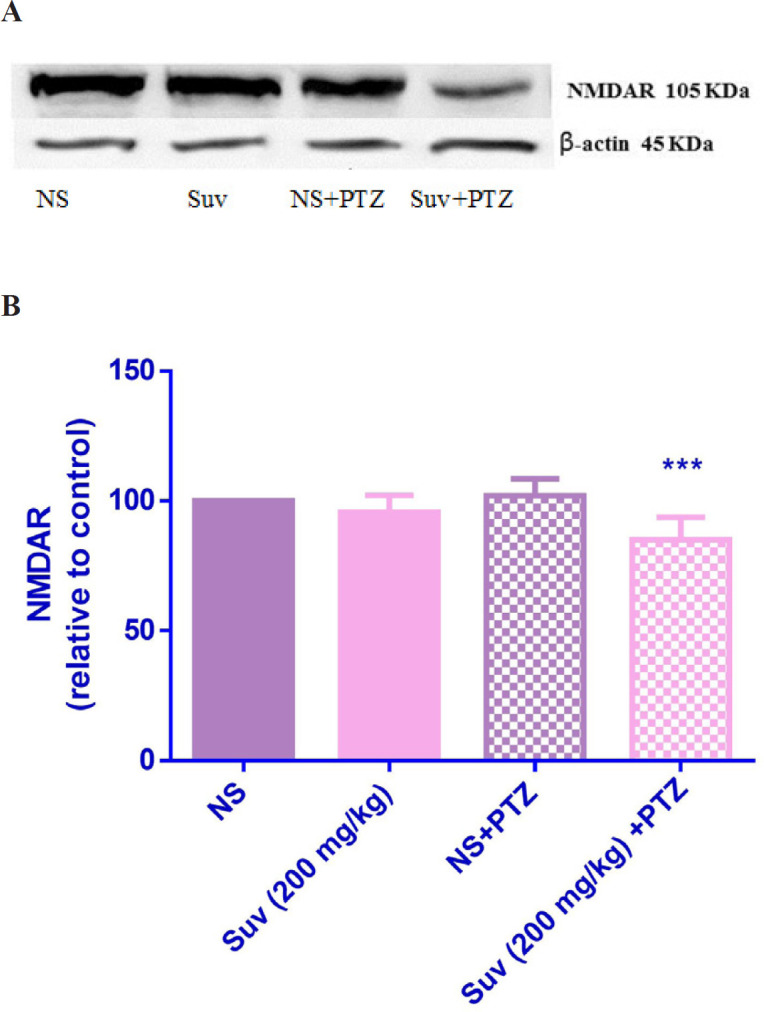
Effect of suvorexant on the protein level of NMDAR in the mice hippocampus. Suvorexant was administrated for seven days. After seizure induction by PTZ, hippocampus was dissected. Representative western blots showing specific bands for NMDAR and β-actin as an internal control. The equal amounts of protein sample (50 µg) obtained from whole hippocampus homogenate were applied in each lane. These bands are representative of four separate experiments. (B) Densitometric data of protein analysis. Data are expressed as the mean ± SEM. ***p < 0.001 vs PTZ+ normal saline. NS (normal saline), SUV (suvorexant), PTZ (pentylenetetrazol).

As shown in Figure 7, in PTZ-induced seizure group, the AMPAR protein level was upregulated in comparison with normal saline treated mice (*p* < 0.01). In PTZ-induced seizure group, which received suvorexant for seven days, the AMPAR protein level was significantly decreased compared to the PTZ-induced seizure group which received normal saline for seven days (*p* < 0.001). In group which received suvorexant without PTZ, the AMPAR protein level was not changed. According to Figure 8, the NMDAR protein level was down regulated in PTZ-induced seizure group, which received suvorexant for seven days compared to PTZ-induced seizure group, receiving normal saline (*p* < 0.001).

## Discussion

The results of the present study indicated that the dose of 200 mg/kg of suvorexant caused significant increases in MCS and GTCS latencies in PTZ model. Flumazenil inhibited the prolongation of seizure latency induced by suvorexant. In MES model, the stretching length of extremities were decreased by suvorexant (100 and 200 mg/kg) and three doses of suvorexant were protected against mortality. The results of western blot showed that the protein level of NMDA and AMPA receptors were decreased by suvorexant. 

A large body of evidences showed that orexin can increase neuronal excitability which leads to epileptic activity (17).  For examples, the intracortical administration of orexin-A and orexin-B induced epileptic activity (9) and increased penicillin-induced seizure (10). Another study revealed that high dose of orexin-A induced seizure-related behaviors after ICV administration in the rats (8). Additionally, tonic–clonic convulsions in epileptic patients caused a reduction in CSF orexin level (18). Accordingly, there are some studies suggesting the protective effects of orexin antagonists against seizure. For examples, SB334867 (selective OX1 receptor antagonist) or TCS (selective OX2 receptor antagonist) significantly increased the latency, decreased the total duration of seizures and the mortality rate in the sleep-deprived rats, exposed to PTZ (7). Furthermore, in another studies protection against seizure-related behaviors of kindled rats has been observed following ICV administration of SB334867 or TCS (19, 20). Similarly, in our research, suvorexant, a dual orexin receptor antagonist, at the dose level of 200 mg/kg significantly increased MCS and GTCS latencies in PTZ model.

It is well known that imbalance between excitatory and inhibitory processes in the brain induces seizures. The main inhibitory and excitatory neurotransmitters in the brain include gamma aminobutyric acid (GABA) and glutamate. Therefore, the modulation of GABA as well as glutamate effects may be considered as the main mechanisms of orexin-induced neuronal excitability (21). According to a study, the hippocampal administration of SB334867 and/or TCS (OX1 receptor or OX2 receptor antagonists) caused a decline of glutamate and an elevation of GABA content in the hippocampus (11). 

In the present study, flumazenil, as an antagonist of BZD site in the GABA_A_-BZD receptor complex, reduced the latency to the first GTCS in suvorexant-treated mice indicating that anticonvulsant effect of suvorexant may be related to interaction with GABA_A_ receptors. 

To study the role of glutamate, the protein expressions of NMDAR and AMPAR were measured in hippocampus in suvorexant-treated mice. Hippocampus is a structure crucially important for learning and memory and highly susceptible to temporal lobe epilepsy (11). Considering the distribution of orexin receptor expression in the hippocampus, it could be suggested that orexin may be involved in the control of seizures in the temporal lobe epilepsy. The results indicated that suvorexant significantly decreased the protein levels of AMPAR and NMDAR in hippocampus of the mice, exposed to PTZ. Hence, the activity of glutamatergic system may be decreased by suvorexant. Our results are in accordance with another study indicating that ICV administration of OX1 receptor antagonist attenuated behavioral seizures and decreased hippocampal glutamate content in rats (22). In addition, the intravenous administration of orexin-A increased glutamate levels in the locus coeruleus (23).

According to data, it could be considered that these neurotransmitters (GABA and glutamate) have important roles in the protective effects of suvorexant on the seizure induced by PTZ.

**Table 1:** Effect of suvorexant on mortality (%) in PTZ model in mice. Fisher test. N = 6

**Group**	**Protection** **against mortality (%)**
Control	33.3
Suvorexant 50 mg/kg	66.6
Suvorexant 100 mg/kg	83.3
Suvorexant 200 mg/kg	83.3
Diazepam 2 mg/kg	100

**Table 2:** Effect of suvorexant on mortality (%) in MES model in mice. Fisher test. N = 6.

**Group**	**Protection** **against mortality (%)**
Control	33.3
Suvorexant 50 mg/kg	66.6
Suvorexant 100 mg/kg	83.3
Suvorexant 200 mg/kg	83.3
Diazepam 2 mg/kg	83.3

## Conclusion

The results of this study demonstrated that suvorexant could exert anticonvulsant activity and in addition to its inhibitory effect on orexin receptors, at least partly, it interacts with GABA_A_ and glutamate receptors.
